# Equilibrium of the intracellular redox state for improving cell growth and l-lysine yield of *Corynebacterium glutamicum* by optimal cofactor swapping

**DOI:** 10.1186/s12934-019-1114-0

**Published:** 2019-04-03

**Authors:** Jian-Zhong Xu, Hao-Zhe Ruan, Xiu-Lai Chen, Feng Zhang, Weiguo Zhang

**Affiliations:** 1 The Key Laboratory of Industrial Biotechnology, Ministry of Education, School of Biotechnology, Jiangnan University, 1800# Lihu Road, Wuxi, 214122 China; 2 State Key Laboratory of Food Science and Technology, School of Biotechnology, Jiangnan University, 1800# Lihu Road, Wuxi, 214122 China

**Keywords:** *Corynebacterium glutamicum*, Glyceraldehyde-3-phosphate dehydrogenase, Isocitrate dehydrogenase, Redox state, Cofactor optimization, l-Lysine production

## Abstract

**Background:**

NAD(H/^+^) and NADP(H/^+^) are the most important redox cofactors in bacteria. However, the intracellular redox balance is in advantage of the cell growth and production of NAD(P)H-dependent products.

**Results:**

In this paper, we rationally engineered glyceraldehyde-3-phosphate dehydrogenase (GAPDH) and isocitrate dehydrogenase (IDH) to switch the nucleotide-cofactor specificity resulting in an increase in final titer [from 85.6 to 121.4 g L^−1^] and carbon yield [from 0.33 to 0.46 g (g glucose)^−1^] of l-lysine in strain RGI in fed-batch fermentation. To do this, we firstly analyzed the production performance of original strain JL-6, indicating that the imbalance of intracellular redox was the limiting factor for l-lysine production. Subsequently, we modified the native GAPDH and indicated that recombinant strain RG with nonnative NADP-GAPDH dramatically changed the intracellular levels of NADH and NADPH. However, l-lysine production did not significantly increase because cell growth was harmed at low NADH level. Lastly, the nonnative NAD-IDH was introduced in strain RG to increase the NADH availability and to equilibrate the intracellular redox. The resulted strain RGI showed the stable ratio of NADPH/NADH at about 1.00, which in turn improved cell growth (μ_max._ = 0.31 h^−1^) and l-lysine productivity (*q*_Lys, max._ = 0.53 g g^−1^ h^−1^) as compared with strain RG (μ_max._ = 0.14 h^−1^ and *q*_Lys, max._ = 0.42 g g^−1^ h^−1^).

**Conclusions:**

This is the first report of balancing the intracellular redox state by switching the nucleotide-cofactor specificity of GAPDH and IDH, thereby improving cell growth and l-lysine production.
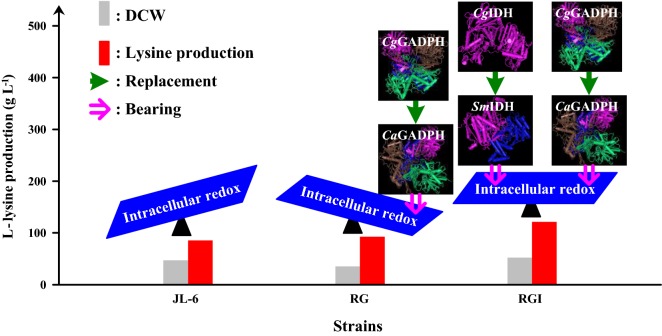

**Electronic supplementary material:**

The online version of this article (10.1186/s12934-019-1114-0) contains supplementary material, which is available to authorized users.

## Background

*Corynebacterium glutamicum* has three major redox systems, i.e., NAD(H/^+^), NADP(H/^+^) and reduced/oxidized glutathione (GSH/GSSG), which are used to adjust the intracellular redox state [1]. They are also involved in other physiological functions, including regulating the energy transfer, controlling the cell life cycle, monitoring the cellular signaling and modulating the microbial virulence [1–3]. NAD(H/^+^) and NADP(H/^+^), as the most important redox cofactors in metabolism, not only provide reducing power for the energy-conserving redox reactions but also act as electron acceptors in the catabolic substrates [4]. However, NAD(H/^+^) is preferentially used in catalyzing substrate oxidation, whereas NADP(H/^+^) is mainly used in catalyzing substrate reduction [5, 6]. Therefore, balancing the oxidation–reduction state of NAD(H/^+^) and NADP(H/^+^) is crucial for both catabolism and anabolism. Some examples have illustrated the importance of redox balance and availability for producing the fine chemicals including amino acids [7–9], vitamins [10, 11] and lipids [12, 13]. l-Lysine, as an essential amino acid for animals and humans, is mainly produced by *C. glutamicum* or its derivatives, which were created by traditional mutagenesis-screening techniques or by genetic engineering [14, 15]. Notably, 4 mol of NADPH must be supplied for 1 mol of l-lysine biosynthesis from oxaloacetate, and thus numerous studies have focused on engineering the metabolism of NADPH for improving l-lysine producing strains [9, 16–19]. For example, genetic modification of the native glyceraldehyde-3-phosphate dehydrogenase (GAPDH) has been proven to improve the l-lysine production because of the increase of NADPH availability [17, 19, 20].

**Fig. 1:**
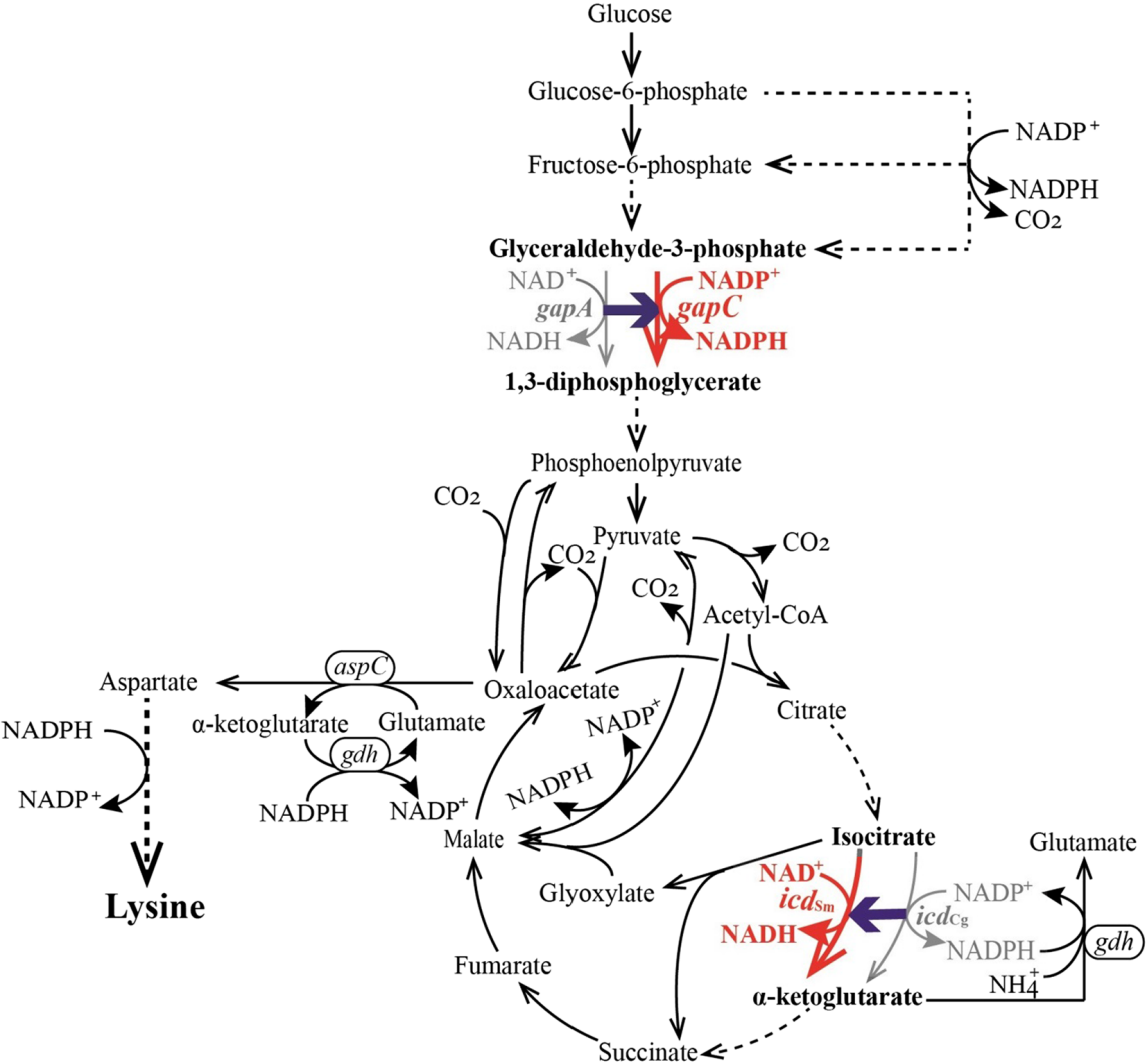
Schematic representation of the l-lysine biosynthesis pathway of *C. glutamicum*. The red font and lines represent the extrinsic routes, whereas the gay font and lines represent the interrupted routes. The blue arrow represents the extrinsic routes replacement procedure

GAPDH catalyzes an essential step in the glycolysis pathway (Fig. 1), which is classified into two types according to their coenzyme specificity, namely, NAD- and NADP-dependent GAPDHs (NAD-GAPDH and NADP-GAPDH) [21]. *C. glutamicum* has two GAPDHs, i.e., GapA (NAD-dependent enzyme) and GapB (NADP^+^-dependent enzyme), but overexpression of GapB-coding gene is bad for the cell growth and l-lysine production [19]. It has been proven that replacement of the native GapA with the phosphorylated NADP-GAPDH from *Clostridium acetobutylicum* [22] or with the non-phosphorylating NADP-GAPDH from *Streptococcus mutans* [19] significantly increased the production of l-lysine. In addition, site-directed modification of the GAPDH binding sites to switch the cofactor specificity can also realize the regeneration of NADPH rather than NADH in the GAPDH-catalyzed step and thus increasing the l-lysine production [20]. It should be noted that the redox pairs are tightly coupled forming a complex redox network in the cell [1]. The excess of NADPH or the insufficient of NADH inhibits the cell growth and carbon consumption. However, many studies are focused mainly on maximizing the level of intracellular NADPH with no regard for NADH (reviewed in Ref. [6]). Therefore, how to balance the intracellular NAD^+^ and NADPH is a critical issue during producing NADPH-dependent products.

In *C. glutamicum*, the reaction catalyzed by the native isocitrate dehydrogenase (*Cg*IDH) in the tricarboxylic acid (TCA) cycle is considered as one of NADPH-generating reaction, because this enzyme displays a 50,000-fold preference for NADP^+^ over NAD^+^ [23]. In contrast, the IDH from *Streptococcus mutans* (*Sm*IDH) shows the strict NAD^+^ dependency [24]. Zhu et al. [25] reported that replacing the *Escherichia coli* IDH (NADP-*Ec*IDH) with the engineered *Ec*IDH^Lys344Asp−Tyr345Ile−Val351Ala^ (NAD-IDH^K344D−Y345I−V351A^) improved the cell growth of the strains with deletion of *udhA* because of the increase of NADH. It should be noted that very few researches focused on *Cg*IDH engineering for breeding the NADPH-dependent products high-yielding strains (e.g., l-lysine high-yielding strains) based on cofactor engineering, to our knowledge. However, the focus of the present study is the replacement of the native NADP-IDH by NAD-IDH from *S. mutans* to switch specificity from NADP^+^ to NAD^+^ in *C. glutamicum*. We provide experimental evidence that replacement of native NAD-GAPDH with NADP-GAPDH as well as replacement of native NADP-IDH with NAD-IDH will balance the intracellular NADH and NADPH, thus improves cell growth and l-lysine production. This is the first report of balancing the intracellular redox state by switching the nucleotide-cofactor specificity of GAPDH combined with IDH, thereby improving cell growth and l-lysine production.

## Results and discussion

### The performance of *C. glutamicum* JL-6 is investigated in fed-batch fermentation in fermenter

**Fig. 2:**
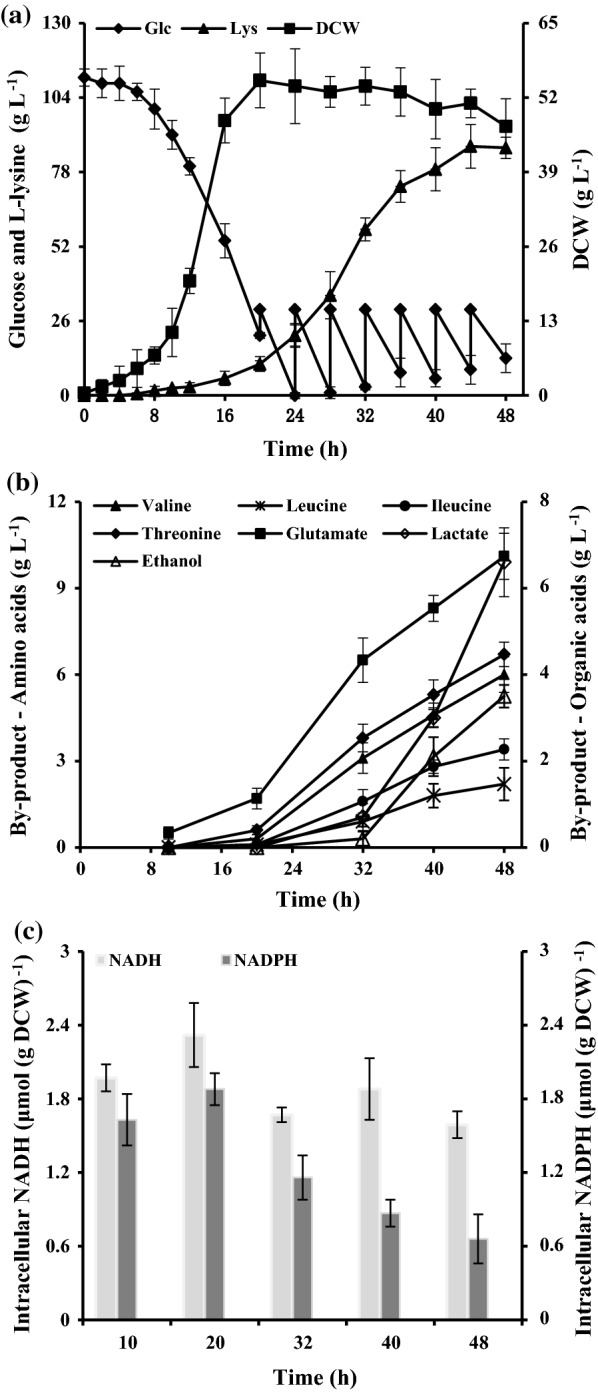
The l-lysine production (filled triangle), cell growth (filled square) and glucose (filled diamond) (**a**), by-products accumulation (**b**), and intracellular level of nucleotide-cofactor (**c**) in strain JL-6 during cultivating in 5-L fermentors. The total glucose consumption of strain JL-6 was 260 g L^−1^. The data represent mean values and standard deviations obtained from three independent cultivations

To get the performance parameters of *C. glutamicum* JL-6, the fermentation was carried out in a 5-L jar fermenter containing 1 L fermentation media. As can be seen from Fig. 2a, the consumed sugar was major used to cell growth and thus resulted in a little l-lysine production during the initial batch phase. The major increase of l-lysine production was achieved during the feeding phase, and continuously increased to a final titer of 85.6 ± 3.7 g L^−1^ (Fig. 2a). Except for l-lysine, the cell excreted about 6.0 ± 0.8 g L^−1^ l-valine, 2.2 ± 0.4 g L^−1^ l-leucine, 3.4 ± 0.6 g L^−1^ l-isoleucine, 6.7 ± 0.3 g L^−1^ l-threonine and 10.1 ± 0.5 g L^−1^ l-glutamine into the broth (Fig. 2b). Same with the l-lysine production, the production of BCAAs (i.e., l-valine, l-leucine, and l-isoleucine) and l-threonine also requires the cofactor NADPH [6]. It should be noted that the rate of glucose uptake and l-lysine production were decreased at the end of fermentation (Fig. 2a). Our previous results [14] and Martinez et al. [26] reports indicated that the GAPDH in glycolysis is inhibited by NADH, and could be a reason of the decrease in the glucose update and l-lysine production. However, NADH-dependent by-products (e.g., lactate and ethanol) concentrations were strongly increased at the end of fermentation (Fig. 2b). This is because intracellular NADH accumulation stimulates the production of NADH-dependent products [27]. In order to confirm the effect of the intracellular NADH/NADPH level on the performance of strain, the changes of intracellular level of NADH or NADPH were monitored over the course of the experiment (Fig. 2c). As might be expected, the intracellular NADH level was at an all-time high, and the NADPH level drastically decreased at the end of fermentation. In addition, the ratio of NADH/NAD^+^ was also at a high level at the logarithmic growth phase (Additional file 1: Table S1). Taken together, these results indicated that *C. glutamicum* JL-6 should be modified based on cofactor engineering to increase l-lysine yield.

### Replacing NAD-GAPDH with NADP-GAPDH increases NADPH production but hampers cell growth

**Fig. 3:**
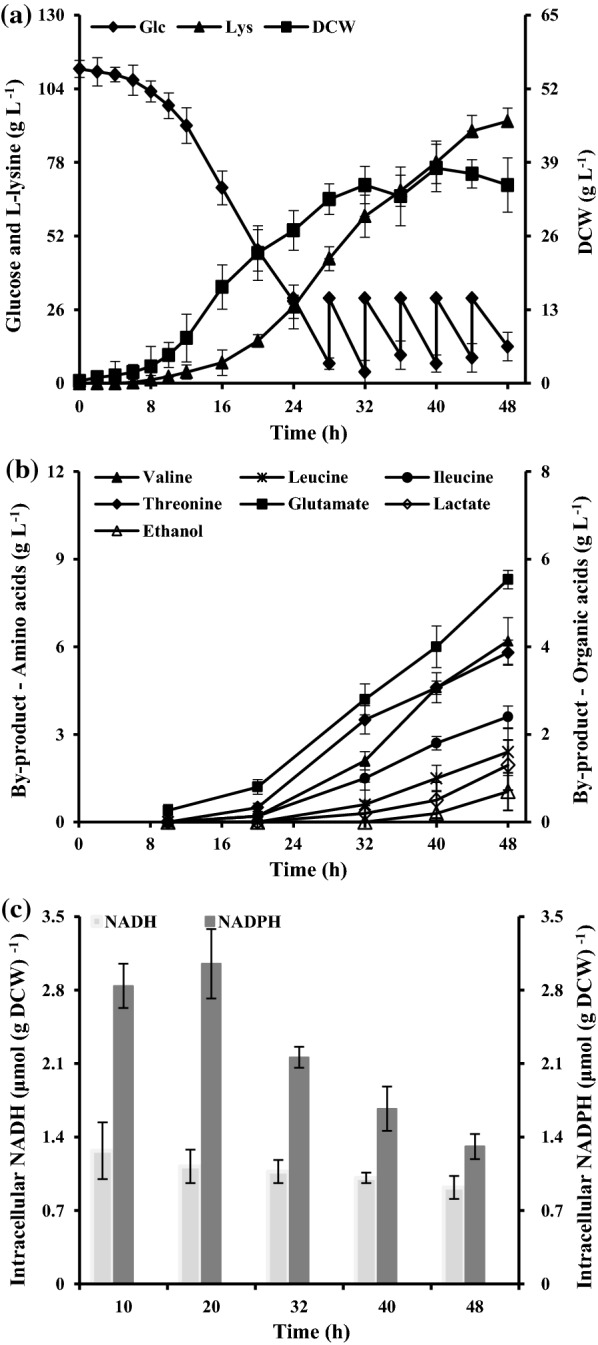
The l-lysine production (filled triangle), cell growth (filled square) and glucose (filled diamond) (**a**), by-products accumulation (**b**), and intracellular level of nucleotide-cofactor (**c**) in strain RG during cultivating in 5-L fermentors. The total glucose consumption of strain RG was 212 g L^−1^. The data represent mean values and standard deviations obtained from three independent cultivations

NAD-GAPDH in *C. glutamicum* was a mainly enzyme for synthesizing NADH [19]. In order to reduce the ratio of NADH/NAD^+^ and to increase the NADPH availability, we replaced the native NAD-GAPDH with NADP-GAPDH from *C. acetobutylicum*, and the performance of recombinant strain JL-6 ∆*gapA::gapC* (i.e., *C. glutamicum* RG) were monitored over the cause of the experiment. The genetically modified strain RG showed no detectable specific NAD-GAPDH activity but exhibited 232.1 ± 15.7 mU (mg^−1^ protein) of NADP-GAPDH, whereas the original strain JL-6 showed 279.5 ± 12.5 mU (mg^−1^ protein) of NAD-GAPDH and no detectable specific NADP-GAPDH activity (Table 1). Accordingly, the NADH level and the ratio of NADH/NAD^+^ was significantly decreased, but the NADPH level and the ratio of NADPH/NADP^+^ was increased in strain RG as compared with strain JL-6 (Fig. 3c and Additional file 1: Table S1). Interestingly, the l-lysine production had not obviously increased along with the NADPH level enhancement (Fig. 3a). This is because strain RG showed a bad cell growth as compared with original strain JL-6, i.e., 35.2 ± 4.5 g L^−1^ and 47.1 ± 3.8 g L^−1^, respectively (Table 2). The similar results were also found in the previous report, and it proved that the NADPH level is too high to imbalance the reducing power [19]. In addition, Zhu et al. [25] pointed out that the cell growth retarded for lack of energy caused by insufficient NADH availability. However, it is heartening that the production strength of strain RG was higher than that of the original strain JL-6, i.e., 2.63 g (g DCW)^−1^ and 1.82 g (g DCW)^−1^, respectively. Moreover, NADH-dependent by-products (e.g., lactate and ethanol) concentrations were strongly decreased at the end of fermentation (Fig. 3b). These results indicated that the production performance of strain RG must be further optimized by balancing the intracellular redox state to increase the cell growth.

**Table 1:** In vitro specific activities of enzymes in genetically modified *C. glutamicum* strains and original strain *C. glutamicum* JL-6 as well as wild-type *C. glutamicum* ATCC13032

*C. glutamicum* strains	Specific activity [mU (mg protein)^−1^]			
	GAPDH		IDH	
	NAD^+^	NADP^+^	NAD^+^	NADP^+^
JL-6	279.5 ± 12.5	–	4.5 ± 1.4	232.5 ± 19.8
RG	–	232.1 ± 15.7	8.9 ± 1.1	216.2 ± 27.3
RGI	–	253.1 ± 21.2	194.5 ± 21.6	–

The assay mixture contained 1 mmol L^−1^ NAD^+^ or NADP^+^ as cofactor, and one unit of enzyme activity corresponds to 1 μmol NADH or NADPH formed per min, which was monitored continuously at A_340_

All data are meaning values of three determinations of three independent experiments with ± SD

–, not detected

**Table 2:** The DCW (24 h or 48 h), l-lysine production (24 h or 48 h), maximal specific growth rate (μ_max._), maximal specific production rate of l-lysine (*q*_Lys, max._), maximal specific substrate uptake rate of glucose (*q*_s, max._) and glucose conversion efficiency (α) of *C. glutamicum* strains

*C. glutamicum* strains	DCW (g L^−1^)		l-Lys yield (g L^−1^)		μ_max_^a^ (h^−1^)	*q*_Lys, max._^b^ (g g^−1^ h^−1^)	*q*_s, max._^c^ (g g^−1^ h^−1^)	α^d^ (%)
	24 h	48 h	24 h	48 h				
JL-6	54.3 ± 6.5	47.1 ± 3.8	21.4 ± 4.1	85.6 ± 3.7	0.23	0.26 ± 0.05	0.80 ± 0.15	32.9
RG	26.3 ± 3.6	35.2 ± 4.5	27.2 ± 6.3	92.5 ± 2.6	0.14	0.42 ± 0.08	0.65 ± 0.32	43.6
RGI	60.3 ± 5.8	52.5 ± 2.8	53.1 ± 7.9	121.4 ± 4.8	0.31	0.53 ± 0.06	0.93 ± 0.24	50.0

All data are meaning values of three determinations of three independent experiments with ± SD

^a^The maximal specific growth rate of cell in the whole fermentation cycle

^b^The maximal specific production rate of l-lysine in the whole fermentation cycle

^c^The maximal specific substrate uptake rate of glucose in the whole fermentation cycle

^d^The conversion rate of glucose into l-lysine after cultivating 48 h

### Replacing NADP-IDH with NAD-IDH balances the intracellular redox state

**Fig. 4:**
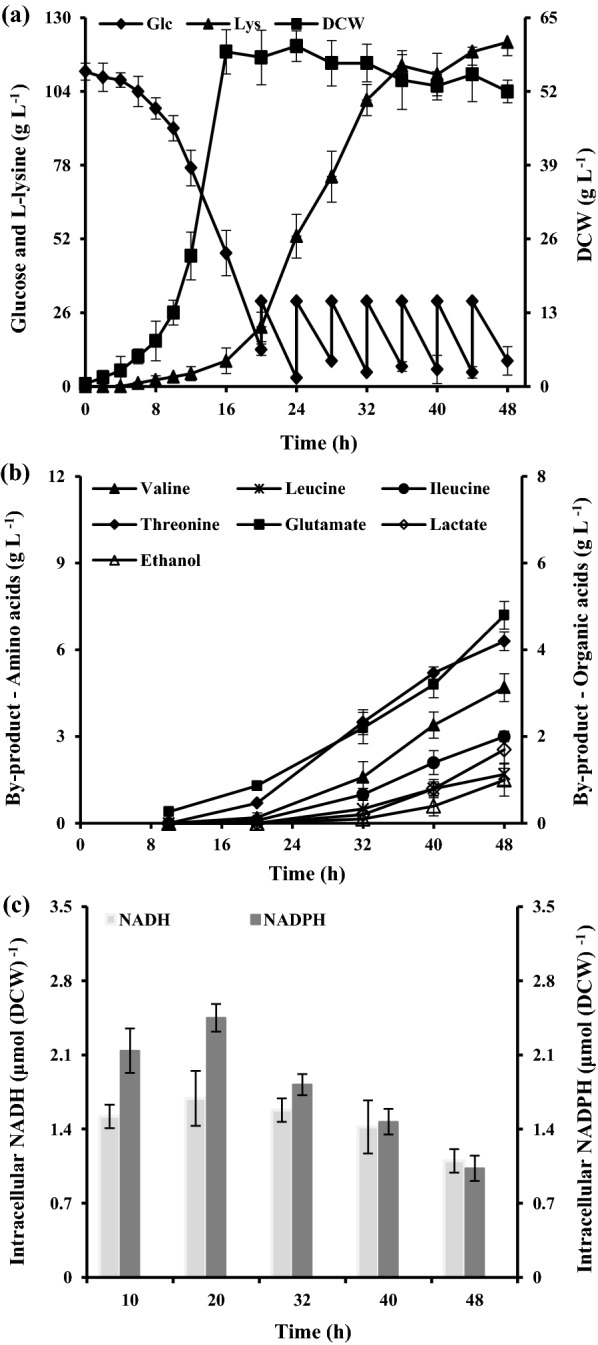
The l-lysine production (filled triangle), cell growth (filled square) and glucose (filled diamond) (**a**), by-products accumulation (**b**), and intracellular level of nucleotide-cofactor (**c**) in strain RGI during cultivating in 5-L fermentors. The total glucose consumption of strain RGI was 264 g L^−1^. The data represent mean values and standard deviations obtained from three independent cultivations

The native *Cg*IDH in *C. glutamicum* is one of NADPH-generating enzyme, whereas the IDH from *S. mutans* (*Sm*IDH) showed strict NAD^+^ dependency [24]. In order to increase the NADH availability and to balance the intracellular redox state, we replaced the native NADP-dependent IDH (NADP-IDH) with NADP-dependent IDH (NAD-IDH) from *S. mutans*, and the performance of recombinant strain RG ∆*icd*_Cg_*::icd*_Sm_ (i.e., *C. glutamicum* RGI) were monitored over the cause of the experiment. The genetically modified strain RGI showed no detectable specific NADP-IDH activity but exhibited 194.5 ± 21.6 mU (mg^−1^ protein) of NAD-IDH, whereas the strain RG showed 216.2 ± 27.3 mU (mg^−1^ protein) of NADP-IDH and 8.9 ± 1.1 mU (mg^−1^ protein) of NAD-IDH (Table 1). Cvitkovitch et al. [24] pointed out that *Sm*IDH is a strict NAD^+^-dependent enzyme, thus no NADP-IDH activity was detected in strain RGI. As was expected, the cell growth was well improved and the glucose consumption rate of strain RGI was higher than that of strain RG (Figs. 3a, 4a; Table 2). The similar results were also found in the previous reports, in which the non-proton-pumping NADH:ubiquinone oxidoreductase (NDH-II) was mutated to switch its nucleotide-cofactor specificity by introducing the D213G mutation in the wild-type enzyme [22] or a spontaneous mutants with improved growth on glucose was isolated [19]. The l-lysine production of strain RGI, of cause, increased significantly with the replacement of IDH. The strain RGI excreted 121.4 ± 4.8 g L^−1^ l-lysine with a space–time yield of 2.53 g L^−1^ h^−1^ and a maximal specific production rate (*q*_Lys, max._) of 0.53 ± 0.06 g g^−1^ h^−1^, whereas the strain RG only accumulated 92.5 ± 2.6 g L^−1^ l-lysine with a space–time yield of 1.93 g L^−1^ h^−1^ and a maximal specific production rate (*q*_Lys, max._) of 0.42 ± 0.08 g g^−1^ h^−1^ (Table 2). These results are in contrast with the reports of Becker et al. [28]; they found that decrease the ICD (also known as IDH) activity is beneficial for improving l-lysine production because of increasing the flux of anaplerotic carboxylation. And for this we modified the *Sm*IDH activity by replacing the promoter of *Sm*IDH-coding gene *icd*_Sm_ in strain RGI. Five classes of promoters (i.e., P_dapA-L1_, P_tac_, P_tac-M_, P_tuf_ and P_sod_) were used in this study. Among them, the P_dapA-L1_ is a weak promoter, whereas the P_tac-M_, P_tuf_ and P_sod_ are the strong promoters in *C. glutamicum* as compared with the P_tac_ [29]. Interestingly, the strains with the higher or the lower NAD-IDH activity were bad for cell growth and l-lysine production as compared with strain RGI (Table 3). We speculated that there would be two reasons: it may be controlled by L-glutamate production or by energy charge. The energy charge is too low to meet the requirement of cell growth, thus bad for l-lysine production during the strains with the lower NAD-IDH activity (Table 3). In contrast, the bad cell growth and the low l-lysine production of strains with the higher NAD-IDH activity may be due to the insufficient supply of L-glutamate. Müller [30] indicated that IDH- and glutamate dehydrogenase-catalytic reactions shape a conjugate pattern in redox reaction. NAD-IDH catalyzes the NADH synthesis rather than NADPH [24], whereas the reaction catalyzed by glutamate dehydrogenase needs NADPH as cofactor [30]. Therefore, the strains with the higher NAD-IDH activity accumulate the little L-glutamate because of the insufficient supply of NADPH. The speculation of these reasons has been validated on examining the concentration of by-products as well as pyridine nucleotides NAD(H/^+^) and NADP(H/^+^)(Fig. 4b, c). The accumulation of L-glutamate decreased to 7.2 ± 0.17 g L^−1^ in strain RGI from 8.3 ± 0.36 g L^−1^ in strain RG (Fig. 4b). Interestingly, the other NADPH-dependent by-products (e.g., l-valine, l-leucine and L- isoleucine) were also decreased to a certain extent, whereas the production of l-threonine was slightly increased (Fig. 4b). This could be that more pyruvate was used to biosynthesize aspartate-family amino acids (e.g., l-threonine and l-lysine) rather than to biosynthesize pyruvate-family amino acids (e.g., l-valine, l-leucine and l-isoleucine). In addition, the strain RGI has certain improvement in the NADH level and the ratio of NADH/NAD (Fig. 4c and Additional file 1: Table S1). More importantly, the NADPH level was just on proper level and the ratio of NADPH/NADH remained fairly constant in the late fermentation stage at about 1.00 in strain RGI (Fig. 4c).

**Table 3:** The DCW, l-lysine production, by-product accumulation, intracellular NADH and NADPH level of modified glutamate dehydrogenase *C. glutamicum* strains

*C. glutamicum* strains	NAD-IDH [mU (mg protein)^−1^]	DCW (g L^−1^)	l-Lysine (g L^−1^)	By-products (g L^−1^)					NADH^a^	NADPH^a^
				l-Valine	l-Threonine	l-Glutamate	Lactate	Ethanol		
RGI (RG/P_tac_ *icd*_Sm_)	243.5 ± 21.6	52.5 ± 2.8	121.4 ± 4.8	4.7 ± 0.48	6.3 ± 0.38	7.2 ± 0.32	1.7 ± 0.24	1.0 ± 0.13	1.52 ± 0.11	2.15 ± 0.24
RG/P_dapA-L1_ *icd*_Sm_	23.2 ± 1.9	39.6 ± 3.5	45.6 ± 3.7	6.1 ± 0.54	5.4 ± 0.33	3.7 ± 0.34	1.2 ± 0.13	0.6 ± 0.05	1.05 ± 0.07	2.17 ± 0.18
RG/P_tac-M_ *icd*_Sm_	321.4 ± 32.8	64.2 ± 5.8	109.7 ± 2.4	4.5 ± 0.27	6.9 ± 0.65	9.1 ± 0.72	1.8 ± 0.08	1.3 ± 0.15	1.86 ± 0.14	2.08 ± 0.13
RG/P_tuf_ *icd*_Sm_	509.4 ± 26.3	34.5 ± 3.7	39.2 ± 2.9	3.2 ± 0.23	4.3 ± 0.45	6.5 ± 0.58	2.1 ± 0.27	1.2 ± 0.08	2.47 ± 0.31	1.98 ± 0.16
RG/P_sod_ *icd*_Sm_	437.6 ± 34.2	49.6 ± 6.3	92.8 ± 8.3	4.3 ± 0.32	6.5 ± 0.48	7.9 ± 0.63	1.9 ± 0.14	1.1 ± 0.11	2.12 ± 0.11	1.93 ± 0.19

All data are meaning values of three determinations of three independent experiments with ± SD

^a^The sampling time is 10 h, and the unit is μmol (g DCW)^−1^

### Changes of carbon flux in strains JL-6, RG and RGI

**Fig. 5:**
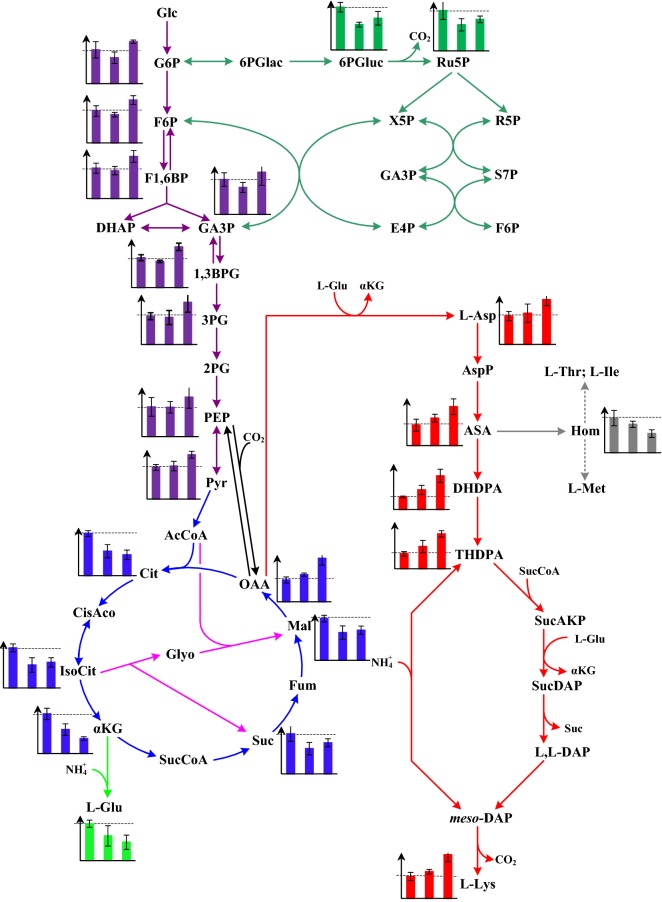
Levels of intermediates involved in l-lysine biosynthesis detected in strains JL-6, RG and RGI. The x-axes represent JL-6, RG and RGI. The y-axes represent the relative abundance of intermediate, which was calculated by normalizing the peak area of metabolite against the total peak area within the sample. *Glc* glucose, *G6P* glucose-6-phosphate, *F6P* fructose-6-phosphate, *F1,6BP* Fructose-1,6-bisphosphate, *DHAP* Dihydroxyacetone phosphate, *GA3P* glyceraldehydes-3-phosphate, *1,3BPG* 1,3-diphosphoglycerate, *3PG* 3-phosphoglycerate, *2PG* 2-phosphoglycerate, *PEP* phosphoenolpyruvate, *Pyr* pyruvate, *AcCoA* acety-CoA, *Cit* citrate, *CisAco* cis-aconitate, *IsoCit*, isocitrate; *α*-*KG*, α-ketoglutarate, *SucCoA*, succinyl-CoA, *Suc* succinate, *Fum* fumarate; *Mal* malate, *OAA* Oxaloacetate, *L*-*Glu* l-glutamate, *6PGlac* 6-phosphoglucono-1,5-lactone, *6PGluc* 6-phosphogluconate, *Ru5P* ribulose-5-phosphate, *X5P* xylulose-5-phosphate, *R5P* ribose-5-phosphate, *S7P* sedoheptulose-7-phosphate, *E4P* erythrose-4-phosphate, *L*-*Asp* l-aspartate, *AspP* l-aspartate phosphate, *ASA* l-aspartate-4-semialdehyde, *DHDPA* L-2,3-dihydrodipicolinate, *THDPA* l-∆^1^-tetrahydrodipicolinate, *SucAKP* l-*N*-succinyl-2-amino-6-ketopimelate, *SucDAP N*-succinyl-l,l-2,6-diaminopimelate, *L,L*-*DAP* l,l-diaminopimelate, *meso*-*DAP meso*-diaminopimelate, *L*-*Lys* l-lysine

As mentioned above, replacing the GAPDH or/and IDH increased the performance of l-lysine fermentation as compared with the original strain JL-6. To study the effects of GAPDH and IDH on l-lysine production, the changes of carbon flux in JL-6, RG and RGI were studied using GC–MS. More than 70 intracellular metabolites showed different levels in JL-6, RG and RGI. Among these 80 metabolites, 22 of them were closely related to l-lysine production in the biosynthetic pathway. To get more detailed view of the changes of carbon flux caused by the replacement of GAPDH and IDH, the relative content of these 22 metabolites were determined in the post-logarithmic phase (Additional file 1: Table S2). Compared with strain JL-6, the contents of intermediates in glycolysis were significantly increased in strain RGI, and conversely these intermediates were slightly decreased in strain RG (Fig. 5). The reasons may be that the imbalance of intracellular redox state disturbed the glucose uptake of strains because of the replacing the GAPDH [19, 25], whereas further replacing the IDH in strain RG leads to balance the intracellular redox state (Additional file 1: Table S1). Interestingly, the carbon flux in pentose phosphate (PP) pathway was lower in strains RG and RGI than in strain JL-6 (Fig. 5). Takeno et al. pointed out that replacement of NAD-GAPDH with NADP-GAPDH shared the function of PP pathway for supplying NADPH [19]. Previous results also indicated that replacement of NAD-GAPDH with NADP-GAPDH reduced the release of CO_2_ from PP pathway, thereby increasing the yield of the target products [17, 31]. However, the contents of intermediates in TCA cycle were dramatically decreased during introduction of NADP-GAPDH or/and NAD-IDH except oxaloacetate, which is the precursor for l-lysine biosynthesis (Fig. 5). Oxaloacetate will turn to the following metabolites in l-lysine biosynthetic pathway with the help of NADPH, thus avoiding the high NADPH in intracellular because 4 mol of NADPH must be supplied for 1 mol of l-lysine biosynthesis [6]. It should be noted that the level of α-ketoglutarate was much lower, whereas the levels of isocitrate, succinate and malate were slightly higher in RGI than in RG. Isocitrate can be catalyzed by isocitrate lyase into glyoxylate bypass that produces succinate and malate [32]. Being short of IDH (Table 1), more isocitrate enter into glyoxylate bypass and that may be the reason for increasing the levels of isocitrate, succinate and malate. Predictably, the contents of intermediates in terminal pathway of l-lysine biosynthesis were dramatically increased in recombination strains. It should be noted that L-homoserine, as by-product of l-lysine production, was much lower in RGI than in JL-6 and RG (Fig. 5).

## Conclusions

In this work, optimal cofactor swapping by introducing different pyridine nucleotide-dependent enzymes has proved to be an ideal choice for maintaining the balance of the intracellular redox state, which is in advantage of the cell growth and production of the NAD(P)H-dependent products, including l-lysine. What’s clear from this study is that replacing the native NAD-GAPDH with NADP-GAPDH and the native NADP-IDH with NAD-IDH balances the intracellular NADH and NADPH levels, thus improves cell growth and l-lysine production.

The replacement of native NAD-GAPDH with NADP-GAPDH in the l-lysine-producing strain JL-6 has been shown to have a positive role on l-lysine production since the 4 mol of NADPH must be supplied for 1 mol of l-lysine biosynthesis. However, it is not beneficial for cell growth because of the shortage of NADH. To alleviate this, we replaced the native NADP-IDH with NAD-IDH from *S. mutans* for the first time to switch the nucleotide-cofactor specificity of IDH, and found that it was beneficial for balancing the intracellular level of NADH and NADPH which in turn improved cell growth and l-lysine production. Fed-batch fermentation of strain RGI resulted in 121.4 ± 4.8 g L^−1^ of l-lysine with a space–time yield of 2.53 g L^−1^ h^−1^ and a maximal specific production rate (*q*_Lys, max._) of 0.53 ± 0.06 g g^−1^ h^−1^. In addition, the other NADPH-dependent by-products (e.g., l-valine, l-leucine and l-isoleucine) were also decreased to a certain extent. However, the production of l-threonine in the strain RGI was higher than that in the strain RG. Therefore, further improvement of l-lysine production with the strain RGI will act to decrease or block-up the accumulation of other NADPH-dependent by-products though the attenuation or knock-out of key gene. Although the efficiency of l-lysine production of strain RGI is relatively low, the l-lysine yield is higher than those reported in literature (Additional file 1: Table S3). Thus, the final strain RGI deserves studying further to increase the production efficiency. To do this, the carbon flux in metabolic network should be optimized in the next work, for example, switching more carbon flux into l-lysine pathway and reducing the carbon loss.

## Methods

### Strains, growth medium and culture conditions

Strains used in this study are listed in Table 4. l-Lysine producing strain *C. glutamicum* JL-6 (*C. glutamicum* AEC^r^ SD^r^ FP^s^ Met^l^) derived from *C. glutamicum* ATCC 13032 after multiple rounds of random mutagenesis, which was resistant to *S*-2-aminoethyl-l-cysteine (AEC^r^) and sulfadiazine (SD^r^), and was sensitive to β-fluoro-pyruvate (FP^s^) as well as a leaky mutant for l-methionine (Met^l^). This strain has been deposited in the China Information Center of Industrial Microbial, and the number is CICIM B1031.

**Table 4:** Strains and plasmids used in this study

Strains and plasmids	Relevant characteristic(s)	References
Strains		
*E. coli* BL21 (DE3)	F^−^ *ompT gal dcm lon hsdS*_*B*_ (r_B_ m_B_) λ(DE3)	Stratagene
*C. glutamicum* JL-6 (i.e., CICIM B1031)	*C. glutamicum* AEC^r^ SD^r^ FP^s^ Met^l^, derived from strain ATCC13032, deposited in China Information Center of Industrial Microbial (CICIM)	CICIM
*C. glutamicum* RG	Replacement of the natural *gapA* gene with the P_tac_-*gapC*-*rrnBT1T2* cassette in strain JL-6 chromosome	This work
*C. glutamicum* RGI (i.e., *C. glutamicum* RG/P_tac_ *icd*_Sm_)	Replacement of the natural *icd*_Cg_ gene with the P_tac_-*icd*_Sm_-*rrnBT1T2* cassette in strain RG chromosome	This work
*C. glutamicum* RG/P_dapA-L1_ *icd*_Sm_	Replacement of the natural *icd*_Cg_ gene with the P_dapA-L1_-*icd*_Sm_-*rrnBT1T2* cassette in strain RG chromosome	This work
*C. glutamicum* RG/P_tac-M_ *icd*_Sm_	Replacement of the natural *icd*_Cg_ gene with the P_tac-M_-*icd*_Sm_-*rrnBT1T2* cassette in strain RG chromosome	This work
*C. glutamicum* RG/P_tuf_ *icd*_Sm_	Replacement of the natural *icd*_Cg_ gene with the P_tuf_-*icd*_Sm_-*rrnBT1T2* cassette in strain RG chromosome	This work
*C. glutamicum* RG/P_sod_ *icd*_Sm_	Replacement of the natural *icd*_Cg_ gene with the P_sod_-*icd*_Sm_-*rrnBT1T2* cassette in strain RG chromosome	This work
Plasmids		
pUC57/*icd*_Sm_	The plasmid with the cassette of *icd*_Sm_ gene (including *Ptac* promoter, *rrnBT1T2* terminator and *icd*_Sm_ operon)	Synthetic
pK18*mobsacB*	Integration vector	[40]
pK18*mobsacB*/*∆gapA::gapC*	Integration vector for replacement of the *gapA* gene by the P_tac_-*gapC*-*rrnBT1T2* cassette	[17]
pK18*mobsacB*/∆*icd*_Cg_*::icd*_Sm_	Integration vector for replacement of the *icd*_Cg_ gene by the P_tac_-*icd*_Sm_-*rrnBT1T2* cassette	This work
pK18*mobsacB*-P_dapA-L1_ *icd*_Sm_	Integration vector for replacement of the P_tac_ promoter of *icd*_Sm_ gene by the *dapA*-*L1* promoter	This work
pK18*mobsacB*-P_tac-M_ *icd*_Sm_	Integration vector for replacement of the P_tac_ promoter of *icd*_Sm_ gene by the *tac*-*M* promoter	This work
pK18*mobsacB*-P_tuf_ *icd*_Sm_	Integration vector for replacement of the P_tac_ promoter of *icd*_Sm_ gene by the *tuf* promoter	This work
pK18*mobsacB*-P_sod_ *icd*_Sm_	Integration vector for replacement of the P_tac_ promoter of *icd*_Sm_ gene by the *sod* promoter	This work

Luria–Bertani (LB) and LBG (LB + 5 g L^−1^ glucose) were used for culturing *E. coli* and *C. glutamicum*, respectively [33]. Epo medium, used for preparing electroporation-competent *C. glutamicum* cells, and LBHIS (LB, Brain Heart Infusion, and sorbitol) medium, used for obtaining recombinant strains, were prepared according to the description reported by van der Rest et al. [34]. Appropriately, *E. coli* was incubated with 50 µg mL^−1^ of kanamycin (Km), and 25 µg mL^−1^ of Km was used to obtain recombinant *C. glutamicum* strains.

The fed-batch fermentations were carried out in a 5-L jar fermenter (BLBio-5GJ-2-H, Bailun Bi-Technology Co. Ltd., Shanghai, China) containing 1 L of medium with an inoculum size of 10% (v/v), from a seed culture grown to ∆OD_562_ = 0.45–0.50 (at a dilution of 25-fold). The seed medium consisted of (per liter): 25 g glucose, 30 g corn steep liquor, 5 g (NH_4_)_2_SO_4_, 1 g KH_2_SO_4_, 0.5 g MgSO_4_∙7H_2_O, 0.25 g l-methionine and 10 g CaCO_3_. The fermentation medium contained (per liter): 80 g glucose, 40 g beet molasses, 30 g corn steep liquor, 50 g (NH_4_)_2_SO_4_, 1.5 g KH_2_SO_4_, 1.0 g MgSO_4_∙7H_2_O, 0.02 g FeSO_4_, 0.02 g MnSO_4_, 0.5 g l-methionine, 0.05 g glycine betaine, 400 μg thiamine-HCl, 800 μg biotin and 2 mL antifoam. The aeration rate, pH, dissolved oxygen levels, and temperature were set as described by Becker et al. [35]. Feed solution, containing 800 g L^−1^ sterile glucose and 400 g L^−1^ (NH_4_)_2_SO_4_, was used to maintain a glucose concentration at 20–30 g L^−1^ by adjusting the feeding rate according to the glucose concentration checked every 4 h.

### DNA manipulations and construction of *C. glutamicum* recombinant strains

The plasmids and oligonucleotides used in this study are listed in Table 4 and Additional file 1: Table S4, respectively. *C. glutamicum* DNA was extracted, and was used as template for the target gene clone using corresponding primers (Additional file 1: Table S4). The cassette of *Sm*IDH-coding gene with *P*_tac_ promoter, *rrnBT1T2* terminator and *Sal*I endonuclease was synthetized by General Biosystems (Anhui), Inc. (Chuzhou, China). The plasmid construction and transformation were performed according to the descriptions of previous reports [17].

The replacements of gene and promoter were executed in *C. glutamicum* chromosome according to the published method [17, 29]. The recombinant plasmids were transferred into *C. glutamicum* competent cell by electroporation, and the recombinant strains were screened on the LBHIS agar plates containing 25 µg mL^−1^ of Km [34]. The recombinants were validated via sequencing by General Biosystems (Anhui), Inc. (Chuzhou, China).

### Enzyme activity assay

The crude enzyme was prepared according to the method reported by Trigoso et al. [36]. After centrifugation at 4 °C for 30 min at 10,000×*g*, the cell-free supernatants were immediately used to determine the enzyme activities. Protein concentrations were determined using the Bradford Protein Quantification Kit (Sangon, Shanghai, China) with bovine serum albumin as standard. The analyses of enzyme activities and protein concentrations were done in triplicate. Specific activity was given as the number of U mg^−1^ of protein. The analyses of enzymes GAPDH and IDH were based on the protocol of Xu et al. [17] and Wang et al. [37], respectively.

### Quantification of intracellular NADH/NAD and NADPH/NADP

The intracellular NADH/NAD and NADPH/NADP were extracted as previously described by Faijes et al. [38], and their concentration were measured using NAD/NADH Quantification Colorimeteric Kit and NADP/NADPH Quantification Colorimeteric Kit (BioVision, Inc., Milpitas, CA) according to the manufacturer’s instructions, respectively.

### Analytical methods

A sample was taken from the shake flasks or fermenter every 2 or 4 h. A half of sample was used to measure the biomass concentration using a spectrophotometer at 600 nm after an appropriate dilution. According to the previous description [17], the correlation factor between dry cell weight (DCW) and OD_600_ was determined as 0.318 (1 OD_600_ = 0.318 g DCW). The other half of sample was diluted 100-fold, and then used to determine the glucose and l-lysine concentration using an SBA-40E immobilized enzyme biosensor (Shandong, China). The intracellular metabolites of different strains were analyzed by gas chromatography–mass spectrometry (GC–MS) according to the previous described [39]. By the end of fermentation, the fermentation liquors were also used to determine the concentration of by-products (including amino acids and organic acids) by high performance liquid chromatography (HPLC) according to the procedure described by Xu et al. [29]. All data were collected from three independent culture samples, and then were analyzed statistically by Student’s *t* test with a two-tailed distribution.

## Additional file

**Additional file 1.** Additional tables.

## Abbreviations

GAPDH: glyceraldehyde-3-phosphate dehydrogenase
IDH: isocitrate dehydrogenases
BCAAs: branch chain amino acids
DCW: dry cell weight
*q*_Lys, max._: maximal specific production rate
AEC: *S*-2-aminoethyl-l-cysteine
SD: sulfadiazine
FP: β-fluoro-pyruvate
GC–MS: gas chromatography–mass spectrometry
HPLC: high performance liquid chromatography
