# Supplementary material for: Equilibrium of the intracellular redox state for improving cell growth and l-lysine yield of Corynebacterium glutamicum by optimal cofactor swapping
Source: Microb Cell Fact. 2019 Apr 3;18:65. doi: 10.1186/s12934-019-1114-0 (PMC6448238; doi:10.1186/s12934-019-1114-0)
Supplement: Supplementary file 1 — Additional file 1. Additional tables. [file 12934_2019_1114_MOESM1_ESM.docx]

**Equilibrium of the intracellular redox state for improving cell growth and L-lysine yield of** ***Corynebacterium glutamicum* by optimal cofactor swapping**

**Jian-Zhong Xu ^*^**

The Key Laboratory of Industrial Biotechnology, Ministry of Education, School of Biotechnology, Jiangnan University, 1800^#^ Lihu Road, WuXi 214122, China; E-mail: [xujianzhong@jiangnan.edu.cn](mailto:xujianzhong@jiangnan.edu.cn)

**Hao-Zhe Ruan**

The Key Laboratory of Industrial Biotechnology, Ministry of Education, School of Biotechnology, Jiangnan University, 1800^#^ Lihu Road, WuXi 214122, China; E-mail: [hz_ruan@sina.com](mailto:hz_ruan@sina.com)

**Xiu-Lai Chen**

State Key Laboratory of Food Science and Technology, School of Biotechnology, Jiangnan University, 1800^#^ Lihu Road, WuXi 214122, China; E-mail: [xiul_chen@sina.com](mailto:xiul_chen@sina.com)

**Feng Zhang**

The Key Laboratory of Industrial Biotechnology, Ministry of Education, School of Biotechnology, Jiangnan University, 1800^#^ Lihu Road, WuXi 214122, China; E-mail: [zhangf_jn@163.com](mailto:zhangf_jn@163.com)

**Weiguo Zhang**

The Key Laboratory of Industrial Biotechnology, Ministry of Education, School of Biotechnology, Jiangnan University, 1800^#^ Lihu Road, WuXi 214122, China; E-mail: [zwgjnedu@sina.cn](mailto:zwgjnedu@sina.cn)

^*^ Corresponding authors:

Jian-Zhong Xu; E-mail: [xujianzhong@jiangnan.edu.cn](mailto:xujianzhong@jiangnan.edu.cn); Tel: +86-510-85329312; Fax: +86-510-85329312

**1 The procedures of integration vectors constructions**

**1.1 The vector pUC57/*icd*_Sm_ artificial synthesis**

The nucleotide sequences of the *Sm*IDH-coding gene *icd*_Sm_ from *Streptococcus mutans* UA159 with the *Ptac* promoter as well as *rrnBT1T2* terminator from the *E. coli-C. glutamicum* suttle expression plasmid pDXW-8 and *Xma*III endonuclease were synthetized by General Biosystems (Anhui), Inc. (Chuzhou, China). The resulted fragments were linked to the plasmid pUC57, and the resulted plasmid was designated as pUC57/*icd*_Sm_*.*

**1.2 The vectors pK18*mobsacB*/*∆icd*_Cg_ and pK18*mobsacB*/∆*icd*_Cg_*::icd*_Sm_ construction**

The *icd*_Cg_ left (*icd*_Cg_-L) and right (*icd*_Cg_-R) arms from *C. glutamicum* were amplified by PCR with the corresponding primer pairs, respectively (Table S1). The resulting fragments were purified by the SanPrep DNA Gel Extraction Kit. The fragments of *icd*_Cg_-L/ *icd*_Cg_-R were purified and digested by suitable restriction enzyme (Table S1), respectively, and then were orderly ligated into pK18*mobsacB* which was similarly digested. The resulting plasmid was designated as pK18*mobsacB*/*∆icd*_Cg_.

For construction of pK18*mobsacB*/*∆icd*_Cg_*::icd*_Sm_, DNA fragment of *icd*_Sm_ was isolated from the plasmid pUC57/*icd*_Sm_ after digested by *Xma*III endonuclease. The fragment was purified and digested by suitable restriction enzyme, and then was ligated into pK18*mobsacB*/*∆icd*_Cg_ which was similarly digested (Table S1), then the resulted plasmid was designated as pK18*mobsacB*/*∆icd*_Cg_*::icd*_Sm_.

**1.3 The integration vector pK18*mobsacB-*P_dapA-L1_ *icd*_Sm_, pK18*mobsacB-*P_tac-M_ *icd*_Sm_, pK18*mobsacB-*P_tuf_ *icd*_Sm_ and pK18*mobsacB-*P_sod_ *icd*_Sm_ construction**

The building process was referred to Figure S1. In PCR1, the left arm of the *icd*_Cg_ gene (i.e., *icd*_CgUp_) was amplified using primers *icd*_Cg_-L-F and *icd*_Cg_-P_dapA_-R or *icd*_Cg_-P_dapA_-R or *icd*_Cg_-P_tac-M_-R or *icd*_Cg_-P_tuf_-R or *icd*_Cg_-P_sod_-R, whereby an overlapping sequence with the *dapA*-L1, *tac-*M, *tuf* and *sod* promoters was artificially added at the 3’-end. In PCR2, the *dapA*-L1, *tac-*M, *tuf* and *sod* promoters were amplified using the corresponding primers, whereby an overlapping sequence with the *icd*_Sm_ gene was artificially added at the 3’-end. In PCR3, the *icd*_Sm_ operon was amplified using primers *icd*_Sm_*-*F and *icd*_Sm_*-*R. In the next step, two DNA fragments from PCR2 and PCR3 are fused in PCR4 with the *dapA*-L1, *tac-*M, *tuf* or *sod* promoter and *icd*_Sm_ operon specific primer sequences used in PCR2 and PCR3, respectively and cleaned DNA from PCR2 and PCR3. Whereafter, the DNA-fragments from PCR1 and PCR4 are fused by PCR5 using cleaned DNA from PCR1 and PCR4 as template DNA and primers *icd*_Cg_-L-F and *icd*_Sm_*-*R. Recognition sites for *Eco*RI and *Sal*I were used for vector-insert-ligation. The fragment was purified and digested by *Eco*RI and *Sal*I, and then was ligated into pK18*mobsacB* which was similarly digested, then the resulting plasmid was designated as pK18*mobsacB-*P_dapA-L1_ *icd*_Sm_, pK18*mobsacB-*P_tac-M_ *icd*_Sm_, pK18*mobsacB-*P_tuf_ *icd*_Sm_ or pK18*mobsacB-*P_sod_ *icd*_Sm_*.*

**2 The procedures of recombinant strains constructions**

**2.1 Construction of *C.glutamicum* JL-6 *∆gapA::gapC* (or *C. glutamicum* RG)**

The integration vector pK18*mobsacB*/∆*gapA::gapC* were transformed into competent *C. glutamicum* JL-6 cells, and then the resulted recombinant strain was designated as *C.glutamicum* JL-6 *∆gapA::gapC* (or *C. glutamicum* RG).

**2.2 Construction of *C. glutamicum* RG ∆*icd*_Cg_*::icd*_Sm_ (or *C. glutamicum* RGI, *C. glutamicum* RG/P_tac_ *icd*_Sm_)**

The integration vector pK18*mobsacB*/∆*icd*_Cg_*::icd*_Sm_ were transformed into competent *C. glutamicum* RG cells, and then the resulted recombinant strain was designated as *C.glutamicum* RG ∆*icd*_Cg_*::icd*_Sm_ (or *C. glutamicum* RGI).

**2.3 Construction of** ***C. glutamicum* RG/P_dapA-L1_ *icd*_Sm_, *C. glutamicum* RG/P_tac-M_ *gdh*, *C. glutamicum* RG/P_tuf_ *icd*_Sm_, and *C. glutamicum* RG/P_sod_ *icd*_Sm_**

The plasmid pK18*mobsacB-*P_dapA-L1_ *icd*_Sm_, pK18*mobsacB-*P_tac-M_ *icd*_Sm_, pK18*mobsacB-*P_tuf_ *icd*_Sm_ or pK18*mobsacB-*P_sod_ *icd*_Sm_ was transformed into *C. glutamicum* RGI, respectively, then the resulting recombinant strain was designated as *C. glutamicum* RG/P_dapA-L1_ *icd*_Sm_, *C. glutamicum* RG/P_tac-M_ *gdh*, *C. glutamicum* RG/P_tuf_ *icd*_Sm_, and *C. glutamicum* RG/P_sod_ *icd*_Sm_, respectively*.*

**Table S1** Comparison of intracellular nucleotides concentrations in original strain and the genetically defined *C. glutamicum* strains.

| *C. glutamicum* strains | NADH ^a^ | NAD^+ a^ | NADH/NAD^+^ | | NADPH ^a^ | NADP^+ a^ | | NADPH/NADP^+^ | |
| --- | --- | --- | --- | --- | --- | --- | --- | --- | --- |
| JL-6 | 1.97 ± 0.21 | 7.64 ± 0.23 | | 0.26 ± 0.03 | 1.63 ± 0.32 | | 1.40 ± 0.11 | | 1.16 ± 0.13 |
| RG | 1.27 ± 0.23 | 7.05 ± 0.26 | | 0.18 ± 0.01 | 2.84 ± 0.21 | | 1.66 ± 0.09 | | 1.71 ± 0.13 |
| RGI | 1.52 ± 0.11 | 7.16 ± 0.24 | | 0.21 ± 0.01 | 2.15 ± 0.24 | | 1.82 ± 0.13 | | 1.18 ± 0.13 |

^a^ The unit is μmol (g DCW)^-1^.

All data are meaning values of three determinations of three independent experiments with ± SD.

**Table S2** The levels of intracellular metabolites involved in L-lysine biosynthesis in JL-6 (Sample A), RG (Sample B) and RGI (Sample C). To ensure the accuracy of the data, five biomass samples of each strain were subjected to GC-MS. Most of the samples were within 94% confidence interval, except sample B2 (dates not show). B2 was judged to be abnormal samples and were omitted from the analysis to ensure the reliability of the results.

| L-Lys | THDPA | DHDPA | Hom | ASA | L-Asp | L-Glu | OAA | Mal | Suc | αKG | IsoCit | Cit | Ru5P | 6PGluc | Pyr | PEP | 3PG | 1,3BPG | GA3P | F1,6BP | F6P | G6P | Metabolite |
| --- | --- | --- | --- | --- | --- | --- | --- | --- | --- | --- | --- | --- | --- | --- | --- | --- | --- | --- | --- | --- | --- | --- | --- |
| 7.173450 | 0.048143 | 0.076312 | 0.011904 | 0.047362 | 0.060565 | 2.139741 | 0.028056 | 0.049056 | 3.330358 | 0.021508 | 0.006043 | 0.043714 | 0.015634 | 0.032934 | 0.508794 | 0.073948 | 0.402342 | 0.84454 | 1.486687 | 0.154034 | 0.182485 | 5.942358 | A1 |
| 7.490556 | 0.045913 | 0.067056 | 0.009056 | 0.050895 | 0.063005 | 2.080072 | 0.027945 | 0.054003 | 3.062050 | 0.183718 | 0.058655 | 0.047032 | 0.019003 | 0.029158 | 0.503445 | 0.060439 | 0.373578 | 0.840054 | 1.518680 | 0.200004 | 0.164357 | 6.113793 | A2 |
| 6.797575 | 0.051673 | 0.065147 | 0.008453 | 0.061086 | 0.058956 | 2.041335 | 0.030067 | 0.050063 | 3.401005 | 0.021544 | 0.058417 | 0.042945 | 0.012572 | 0.035073 | 0.550685 | 0.066906 | 0.430056 | 0.785749 | 1.390457 | 0.124592 | 0.240053 | 6.876436 | A3 |
| 7.00765 | 0.050676 | 0.073005 | 0.007245 | 0.045896 | 0.060075 | 2.070135 | 0.026989 | 0.049375 | 3.309687 | 0.020456 | 0.061678 | 0.043979 | 0.018789 | 0.028077 | 0.529658 | 0.075451 | 0.390367 | 0.794564 | 1.456964 | 0.137215 | 0.236312 | 6.576985 | A4 |
| 7.800064 | 0.043998 | 0.065845 | 0.008805 | 0.056002 | 0.066078 | 2.003412 | 0.027843 | 0.047966 | 3.094053 | 0.022007 | 0.056846 | 0.044175 | 0.017052 | 0.027918 | 0.510095 | 0.087957 | 0.407524 | 0.810439 | 1.456964 | 0.137215 | 0.236312 | 6.576985 | A5 |
| 8.897564 | 0.071167 | 0.108456 | 0.007052 | 0.065045 | 0.063469 | 1.341345 | 0.035076 | 0.033068 | 1..839976 | 0.013007 | 0.035894 | 0.022795 | 0.009032 | 0.020574 | 0.521085 | 0.080503 | 0.394593 | 0.705686 | 1.157485 | 0.140513 | 0.180032 | 4.556875 | B1 |
| 8.713434 | 0.057763 | 0.110143 | 0.009754 | 0.075093 | 0.077696 | 1.510146 | 0.038147 | 0.037005 | 2.101006 | 0.10263 | 0.031524 | 0.028917 | 0.011659 | 0.017011 | 0.574519 | 0.072495 | 0.372485 | 0.740023 | 1.115682 | 0.118976 | 0.200253 | 5.294568 | B3 |
| 9.200565 | 0.083940 | 0.084676 | 0.005939 | 0.069050 | 0.070055 | 1.29046 | 0.034102 | 0.032057 | 1.900043 | 0.015097 | 0.036606 | 0.027873 | 0.011802 | 0.019837 | 0.508658 | 0.060454 | 0.409437 | 0.670586 | 1.099568 | 0.150057 | 0.159805 | 4.456783 | B4 |
| 8.58543 | 0.073067 | 0.130013 | 0.007083 | 0.071045 | 0.060677 | 1.400087 | 0.032968 | 0.031058 | 2.287990 | 0.011801 | 0.033787 | 0.023192 | 0.010415 | 0.015075 | 0.559865 | 0.074973 | 0.350045 | 0.710024 | 1.130586 | 0.161013 | 0.170594 | 5.026784 | B5 |
| 16.05642 | 0.126632 | 0.170315 | 0.005505 | 0.10075 | 0.097007 | 1.000134 | 0.053968 | 0.039055 | 2.407891 | 0.008546 | 0.038694 | 0.020653 | 0.015417 | 0.021768 | 0.706855 | 0.099456 | 0.620547 | 1.104358 | 1.731754 | 0.251507 | 0.240458 | 7.932978 | C1 |
| 13.86343 | 0.127065 | 0.190007 | 0.004156 | 0.082576 | 0.090957 | 1.007677 | 0.058095 | 0.036783 | 2.500062 | 0.007856 | 0.041004 | 0.023643 | 0.011073 | 0.029647 | 0.745779 | 0.095154 | 0.609843 | 1.098431 | 1.811587 | 0.216974 | 0.281325 | 7.383497 | C2 |
| 16.00873 | 0.089973 | 0.184556 | 0.006043 | 0.093265 | 0.098054 | 0.990475 | 0.061006 | 0.034089 | 2.705614 | 0.007545 | 0.037534 | 0.019795 | 0.012552 | 0.017005 | 0.765003 | 0.103465 | 0.603445 | 1.050789 | 1.807699 | 0.199845 | 0.300448 | 8.000436 | C3 |
| 12.50468 | 0.100003 | 0.215647 | 0.004624 | 0.110683 | 0.102006 | 1.100046 | 0.053047 | 0.033877 | 2.360034 | 0.007899 | 0.034982 | 0.020631 | 0.012023 | 0.023013 | 0.680962 | 0.087746 | 0.550034 | 1.130596 | 1.680031 | 0.230054 | 0.258745 | 8.104382 | C4 |
| 14.03566 | 0.119057 | 0.169319 | 0.004743 | 0.100032 | 0.100125 | 1.050735 | 0.055039 | 0.037082 | 2.560034 | 0.007103 | 0.040048 | 0.025004 | 0.013756 | 0.021613 | 0.739869 | 0.098005 | 0.595676 | 1.120343 | 1.801264 | 0.231315 | 0.270334 | 7.700057 | C5 |

**Table S3** Overview on the L-lysine producing strains of *Corynebacterium glutamicum* based on the different strategies for increasing NADPH regeneration.

| *C. glutamicum* strain | Strategies | Final titer  (g L^-1^) | Carbon yield  (%) | Productivity  (g L^-1^ h^-1^) | Reference |
| --- | --- | --- | --- | --- | --- |
| JL-6 | / ^a^ | 85.6 | 33 | 1.78 | This work |
| RG | Replacement of the native GAPDH with NADP-GAPDH | 92.5 | 44 | 1.93 | This work |
| RGI | Replacement of the native GAPDH and IDH with NADP-GAPDH and NAD-IDH, respectively | 121.4 | 46 | 2.53 | This work |
| DSM5715 Pgi mutant | Disruption of the *pgi* gene | 7.19 ^b^ | 24 | 0.15 | [1] |
| DM1730/pEKEx2-pntAB | Hetero-expression of PntAB, a membrane-bound transhydrogenase | 15.66 ^b, c^ | 17 | 0.22 | [2] |
| DM1729(pEKEx3-*ppnK*) | Overexpression of PpnK, an polyphosphate/ATP-dependent NAD kinase | 5.4 ^b, c^ | 11 | - ^d^ | [3] |
| LYS-12 | Enhancement of oxidative pentose phosphate pathway | 120 | 55 | 4.0 | [4] |
| RE2A^iol^/pCAK311 | Replacement of the native GAPDH with NADP-GAPDH | 12.4 ^b, c^ | 62 | 0.31 | [5] |
| gapA M2 | Rational design of the coenzyme specificity of GAPDH | 4.5 ^b^ | 0.15 | 0.23 | [6] |
| JL-6 Δ*dapB::Ec-dapB*^C115G,G116C^ | Replacement of the native dihydrodipicolinate reductase with the mutated dihydrodipicolinate reductase from *Escherichia coli* | 117.3 | 44 | 2.93 | [7] |

^a^ It is the original strain.

^b^ Achieved in shake‑flask fermentation.

^c^ Estimated from reference.

^d^ No data was given.

**Table S4** The oligonucleotides used in this study

| Oligonucleotide | | 5’→3’ sequence ^a^ | Cleavage sites | | | Purposes |
| --- | --- | --- | --- | --- | --- | --- |
| *gapA*-F | ATGACCATTCGTGTTG | | | - | PCR for the *C. glutamicum gapA*operon | |
| *gapA*-R | TTAGAGCTTGGAAGCTAC | | | - |  |  |
| *gapC*-F | ATGGCAAAGATAGCTATTAATG | | | - | PCR for the *Clostridium acetobutylicum gapC* operon | |
| *gapC*-R | CTATTTTGCTATTTTTTGCAAAG | | | - |  |  |
| *icd*_cg_-F | ATGGCTAAGATCATCTGGAC | | | - | PCR for the *C. glutamicum icd* operon | |
| *icd*_cg_-R | TTACTTCTTCAGTGCGAACG | | | - |  |  |
| *icd*_Sm_-F | ATGGCAGAAAAAGTAAG | | | - | PCR for the *Streptococcus mutans icd* operon | |
| *icd*_Sm_-R | ACGCGGATCCCTATAAATAAGTCAATAG | | | *Sal*I |  |  |
| *icd*_cg_-L-F | CGGAATTCCGGCCGCAAGCACGGTG | | | *Eco*RI | PCR for the *C. glutamicum icd* left arm, *icd*_cg_-L | |
| *icd*_cg_-L-R | ACGCGGATCCCTCGTCGGTGGTGGCG | | | *Sal*I |  |  |
| *icd*_cg_-R-F | ACGCGGATCCGAGTTCCTCGCACTGGCTG | | | *Sal*I | PCR for the *C. glutamicum icd* right arm, *icd*_cg_-R | |
| *icd*_cg_-R-R | CCCAAGCTTATGATGTCTTTGGCTTCGC | | | *Hin*dIII |  |  |
| *icd*_cg_-P_dapA_-R | **GCTACCTGCAGCTTTCTTAAACATTCTACC**CTCGTCGGTGGTGGCG | | | *-* | PCR for the *icd*_cg_-L with the P_dapA-L1_ homologous sequence | |
| *icd*_cg_-P_tac-M_-R | **GTTATTTTGACAGACGAATGTATTTGTCA**CTCGTCGGTGGTGGCG | | | *-* | PCR for the *icd*_cg_-L with the P_tac-M_ homologous sequence | |
| *icd*_cg_-P_tuf_-R | **CACTTACCCTACGCGCCTACTGACACGCT**CTCGTCGGTGGTGGCG | | | *-* | PCR for the *icd*_cg_-L with the P_tuf_ homologous sequence | |
| *icd*_cg_-P_sod_-R | **GTGGCAGGCATCCTGTTTTAGAAAATC**CTCGTCGGTGGTGGCG | | | *-* | PCR for the *icd*_cg_-L with the P_sod_ homologous sequence | |

**Table S4** The oligonucleotides used in this study (Continued)

| Oligonucleotide | | 5’→3’ sequence ^a^ | Cleavage sites | | | Purposes |
| --- | --- | --- | --- | --- | --- | --- |
| P_dapA-L1_-F | **GGTAGAATGTTTAAGAAAGCTGCAGGTAGC** | | | ***-*** | PCR for the *dapA-L1* promoter, P_dapA-L1_ used for *icd*_Sm_ modification | |
| P_dapA-L1_-R | **CTTCTTCAAAACTTACTTTTTCTGCCAT**AGAGTTCAAGGTTACCTTC | | | *-* |  |  |
| P_tac-M_-F | **GGTAGAATGTTTAAGAAAGCTGCAGGTAGC** | | | ***-*** | PCR for the P_tac-M_ promoter, P_tac-M_ used for *icd*_Sm_ modification | |
| P_tac-M_ -R | **CTTCTTCAAAACTTACTTTTTCTGCCAT**AGAGTTCAAGGTTACCTTC | | | *-* |  |  |
| P_tuf_-F | **GGTAGAATGTTTAAGAAAGCTGCAGGTAGC** | | | ***-*** | PCR for the P_tuf_ promoter, P_tuf_ used for *icd*_Sm_ modification | |
| P_tuf_-R | **CTTCTTCAAAACTTACTTTTTCTGCCAT**AGAGTTCAAGGTTACCTTC | | | *-* |  |  |
| P_sod_-F | **GGTAGAATGTTTAAGAAAGCTGCAGGTAGC** | | | ***-*** | PCR for the P_sod_ promoter, P_sod_ used for *icd*_Sm_ modification | |
| P_sod_-R | **CTTCTTCAAAACTTACTTTTTCTGCCAT**AGAGTTCAAGGTTACCTTC | | | *-* |  |  |

^a^ Cleavage sites are underlined; Sequence in bold: homologous recombination sequences; -: No cleavage sites.

**Fig. S1.** Strategy used for construction of recombinant plasmid used for the replacement of promoter of the target gene. *T* represents the modified gene; *T_Up_* represents the upstream regions of the modified gene; *P* represents the promoter; *P1-P6* represents the primers; *E1* and *E2* represent the restriction enzymes. The lines in the same color represent the homologous sequence.

**Supplementary References**

[1] Marx A, Hans S, Mockel B, Bathe B, de Graaf AA: **Metabolic phenotype of phosphoglucose isomerase mutants of *Corynebacterium glutamicum***. *J Biotechnol* 2003, **104**: 185-197.

[2] Kabus A, Georgi T, Wendisch VF, Bott M: **Expression of the *Escherichia coli pntAB* genes encoding a membrane-bound transhydrogenase in *Corynebacterium glutamicum* improves L-lysine formation**. *Appl Microbiol Biotechnol* 2007, **75**:47-53.

[3] Lindner SN, Niederholtmeyer H, Schmitz K, Schoberth SM, Wendisch VF: **Polyphosphate/ATP-dependent NAD kinase of *Corynebacterium glutamicum*: biochemical properties and impact of *ppnK* overexpression on lysine production**. *Appl Microbiol Biotechnol* 2010, **87**:583-593.

[4] Becker J, Zelder O, Hafner S, Schroder H, Wittmann C: **From zero to hero-Design-based systems metabolic engineering of *Corynebacterium glutamicum* for L-lysine production.** *Metab Eng* 2011, **13:**159-168.

[5] Takeno S, Hori K, Ohtani S, Mimura A, Mitsuhashi S, Ikeda M: **L-Lysine production independent of the oxidative pentose phosphate pathway by *Corynebacterium glutamicum* with the *Streptococcus mutans* *gapN* gene.** *Metab Eng* 2016, **37:**1-10.

[6] Bommareddy RR, Chen Z, Rappert S, Zeng AP: **A *de novo* NADPH generation pathway for improving lysine production of *Corynebacterium glutamicum* by rational design of the coenzyme specificity of glyceraldehyde 3-phosphate dehydrogenase.** *Metab Eng* 2014, **25:**30-37.

[7] Xu JZ, Yang HK, Liu LM, Wang YY, Zhang WG: **Rational modification of *Corynebacterium glutamicum* dihydrodipicolinate reductase to switch the nucleotide-cofactor specificity for increasing L-lysine production**. *Biotechnol Bioeng* 2018, **115**:1764-1777.
